# Natural Variation Identifies Multiple Loci Controlling Petal Shape and Size in *Arabidopsis thaliana*

**DOI:** 10.1371/journal.pone.0056743

**Published:** 2013-02-13

**Authors:** Mary C. Abraham, Chanatip Metheetrairut, Vivian F. Irish

**Affiliations:** 1 Department of Molecular, Cellular and Developmental Biology, Yale University, New Haven, Connecticut, United States of America; 2 Department of Ecology and Evolutionary Biology, Yale University, New Haven, Connecticut, United States of America; Universidad Miguel Hernández de Elche, Spain

## Abstract

Natural variation in organ morphologies can have adaptive significance and contribute to speciation. However, the underlying allelic differences responsible for variation in organ size and shape remain poorly understood. We have utilized natural phenotypic variation in three *Arabidopsis thaliana* ecotypes to examine the genetic basis for quantitative variation in petal length, width, area, and shape. We identified 23 loci responsible for such variation, many of which appear to correspond to genes not previously implicated in controlling organ morphology. These analyses also demonstrated that allelic differences at distinct loci can independently affect petal length, width, area or shape, suggesting that these traits behave as independent modules. We also showed that *ERECTA* (*ER*), encoding a leucine-rich repeat (LRR) receptor-like serine-threonine kinase, is a major effect locus determining petal shape. Allelic variation at the *ER* locus was associated with differences in petal cell proliferation and concomitant effects on petal shape. *ER* has been previously shown to be required for regulating cell division and expansion in other contexts; the *ER* receptor-like kinase functioning to also control organ-specific proliferation patterns suggests that allelic variation in common signaling components may nonetheless have been a key factor in morphological diversification.

## Introduction

Within a species, individuals are remarkably consistent in their shape and size, yet differences in scale are often the most striking observations when comparisons between species are made [1]. Individual organs also typically grow to a consistent species-specific size. During organogenesis, randomly oriented cell divisions and cell expansion enlarge tissues, oriented cell divisions promote directional growth, and cell-cell interactions and long range signaling processes regulate overall size [2]. The observation of compensatory growth in which variation in cell number or cell volume nonetheless results in a consistent organ size, has led to the suggestion that size is controlled at the whole organ level [2], [3].

In plants, both morphogen gradients and localized cell-cell interactions have been postulated to be involved in regulating organ shape and size [4], [5]. In addition, mechanical feedback controls are required to ensure the development of an appropriate organ form [6]–[8]. Despite these observations, the genes and molecular processes underpinning these regulatory controls have in large part remained unidentified.

The *Arabidopsis thaliana* petal has a relatively simple laminar morphology and is an ideal plant system with which to analyze organ growth control [9]. Petal size and shape is remarkably consistent within a given ecotype [10], [11], in contrast to leaves which show much higher variability under different environmental conditions [12]. Early phases of *Arabidopsis thaliana* petal growth depend on cell division that proceeds in a basipetal fashion, while later stages appear to depend predominantly on cell expansion [13]–[16]. Cell expansion accounts for most of the increase in petal mass and is mainly caused by the vacuolar uptake of water [17]. The epidermal L1 cell layer has been shown to control overall petal size and shape, pointing to a role for directional interlayer cell-cell interactions in regulating petal form [18].

Although the molecular basis for growth control at the organ level is still poorly understood, some genes have been identified that appear to have critical roles in regulating petal growth and form. Cell proliferation inhibitors including *BIG BROTHER* (*BB*), encoding an E3 ubiquitin ligase, and *DA1*, encoding a ubiquitin acceptor, can affect overall organ size, implicating proteolytic cleavage as an important mechanism in regulating this process [19], [20]. *JAGGED* (*JAG*) and *AINTEGUMENTA* (*ANT*), putative transcriptional regulators, promote petal cell proliferation and regulate different aspects of organ size and shape [15], [21]–[23]. At later stages of petal development, *miR319a* acts to regulate the accumulation of class II *TCP* gene products which are required for cell proliferation and thus control overall petal growth [24]–[26]. Regulation of cell expansion has also been shown to be important in defining petal size; for example, *BIG PETALp* (*BPEp*) encodes an auxin- and jasmonate-responsive transcription factor that limits petal cell expansion and has a concomitant effect on petal size [27]–[29]. In addition, long-range signals may be acting to control overall size of an individual organ. *KLUH/CYP78A5*, encoding a cytochrome p450, has been suggested to globally control organ size through regulation of a long-range mobile signal that modulates the extent of cell proliferation in later stage petals and other organs [30]–[33].

The genetic and molecular basis of quantitative traits such as organ size or shape can be characterized through whole-genome analyses to identify quantitative trait loci (QTL) that underlie phenotypic variation. Different *Arabidopsis thaliana* ecotypes can have quite distinct petal forms that can in turn influence fitness [11]. Despite this variation, only a few studies using intraspecific comparisons of segregating populations of *Arabidopsis thaliana* have been carried out to identify QTL affecting floral organ shape or size [34], [35]. However, the corresponding genes have not yet been molecularly identified and so it is difficult to determine whether the QTL identified in these studies correspond to known growth control genes. QTL analyses can identify naturally occurring alleles that would not be easily recovered in mutant screens [36], therefore a QTL approach can provide new insights into the mechanistic control of organ form.

In this study we utilized natural variation in petal form to identify multiple loci responsible for different aspects of petal shape and size. We utilized both recombinant inbred lines (RILs) [37], as well as advanced intercross recombinant inbred lines (AI-RILs) [38], to define 23 QTLs associated with petal size and shape. The use of RILs from multiple ecotypes allowed us to assess a wider range of polymorphic variation. Furthermore, by analyzing petal-specific aspects of organ growth—controlling for general variation in floral organ length, width or area—we were able to identify QTL that had predominant effects on these petal traits. We recovered a number of distinct QTL for different aspects of petal form, indicating that the genetic regulation of each of these traits can occur in an independent manner. Surprisingly, the loci we identified do not, for the most part, map to known growth control loci or to several previously identified QTL implicated in regulating organ form [34]. We utilized near isogenic lines (NILs) and transgenic complementation to show that one major effect QTL controlling petal shape maps to a single genetic locus, *ERECTA* (*ER*). We also show that *ER* acts to control petal shape through coordinately controlling cell proliferation across the petal. *ER* encodes a leucine-rich repeat (LRR) receptor-like serine/threonine kinase [39] and so likely plays a role in mediating intercellular signaling events critical for regulating petal shape.

## Materials and Methods

### Plant Material and Growth Conditions

*Arabidopsis* RIL population seeds were obtained from the *Arabidopsis* Biological Resource Center (ABRC) (Ohio State University, USA). These were CS39289 (Col-0×Est-1) and CS1899 (L*er*-0×Col-4). Other seeds obtained from the ABRC included Salk_074642C (*ult1-3* [40]), CS1298 (Landsberg, La-0), CS28796 (Vancouver-0, Van-0), CS76349 (Vezzano-2), CS76352 (Rovero), CS76353 (Altenburg), CS76354 (Mitterberg-1), CS76356 (Castel Feder), CS76358 (Bozen-1), CS76368 (Aposto-1), CS3378 (*er-1*), CS3401 (*er-2*), CS89504 (*er-105*), CS3913 (*er-109*), and CS3924 (*er-120*).

Plants were grown on a mixture of fertilized potting soil and vermiculite (1∶2), and following seed germination treatment of three days at 4°C in the dark, plants were subsequently grown under the following conditions: 22°C (day) and 18°C (night) and long days photoperiod (16 hours light, 8 hours darkness) in a Conviron growth chamber.

### Microscopy

Floral buds were dissected using a stereomicroscope to remove all 4 petals at fully reflexed petal stage 13 [13] along with all 4 sepals and placed on a drop of water or 0.2% agar on a glass slide and photographed with a Leica camera. Only buds between bud positions 5 and 16 on the main stem were used. Measurements were taken of petal and sepal width, length, area, and shape (length/width), and data analyzed using Tomatoanalyzer [41] or MetaMorph (Version 7.6.1.0 Molecular Devices http://www.moleculardevices.com). For the QTL studies, sepals and petals from 5 buds were scored per RIL, in two biological replicates. 98 RILs were scored for the L*er*-0×Col-4 population, and 113 RILs from the Col-0×Est-1 population were used.

To estimate petal cell size in the blade and the claw, stage 13 petals from Col-0 wild type and *er-120* mutant lines were imaged using light microscopy with a Zeiss compound microscope and the number of cells in randomly sampled areas in the blade or the claw were recorded using ImageJ [42] or Metamorph (Molecular Devices, Sunnyvale, CA) software. The size of the sampling areas were 50×50 and 100×100 microns for the blade and claw respectively. For the blade measurements, at least 6 independent regions were sampled per petal and a minimum of 4 petals per line assessed. For the claw measurements, at least 3 independent regions were sampled per petal and a minimum of 3 petals per line was assessed. For the measurements of the proportion of blade and claw length in the same petal, stage 13 petals were photographed. The transition point between blade and claw was defined as the midpoint between all green (claw) and all white (blade) cells.

### QTL and Statistical Analysis

For QTL analysis individual RILs were phenotyped as described. The genetic map for the L*er*-0×Col-4 RIL population came from [43]. The genetic map for Col-0×Est-1 was obtained from Christopher Schwartz and Detlef Weigel, MPI for Developmental Biology, Spemannstraße 37–39, D-72076 Tübingen, Germany.

QTLs were calculated by Composite Interval Mapping [44] using the program QTL Cartographer version 2.5 (Wang S., C. J. Basten, and Z.-B. Zeng, 2011, Windows QTL Cartographer 2.5. Department of Statistics, North Carolina State University, Raleigh, NC. http://statgen.ncsu.edu/qtlcart/WQTLCart.htm), using model 6, a window size for excluding background QTL of 10 cM, and a walk speed of 2 cM. Genome-wide likelihood of odds thresholds (LOD) [45] for each trait were estimated in QTL Cartographer using 1000 random permutations at the 0.05 significance level as previously described [46] and estimates of percentage variance were calculated using QTL Cartographer.

Broad Sense Heritability, the ratio between the genetic variance and the total phenotypic variance (between and among RILs) was calculated using ANOVA using the formula H^2^ = σ^2^_g_/[σ^2^_g_+σ^2^_e_/n] where σ^2^_g_ is the genetic variance, σ^2^_e_ is the environmental variance and n is the number of replicates [47]. For the ANOVA, we scored 5 independent buds from 5 plants for each RIL; for the Col-4×L*er*-0 population we scored 490 buds and for the Col-0×Est-1 population we scored 565 buds. Broad sense heritability was calculated using the entire dataset for each RIL.

For fine mapping of the *CLS1* QTL, Stepped Aligned Inbred Recombinant Strains (STAIRS) for chromosome II were obtained from the Kearsey lab [48]. The genetic markers nearest to the boundaries between Col and L*er* were AT2G17590 (STAIRS line 1215); AT2G25295 (STAIRS line 1217); AT2G14900 (STAIRS line 1233); AT2G18350 (STAIRS line 1240); AT2G23030 (STAIRS line 1262); AT2G30500 (STAIRS 1274); AT2G14900 (STAIRS line 1301); AT2G25295 (STAIRS line 1314). The confidence intervals were calculated according to [49].

### Polymorphism analyses

Polymorphism data were obtained from Polymorph (http://polymorph.weigelworld.org/cgi-bin/webapp.cgi), GBrowse (http://gmod.org/wiki/GBrowse) and the Arabidopsis 1001 Genomes Project (http://signal.salk.edu/atg1001/3.0/gebrowser.php).

## Results

### Natural Variation in Petal Shape and Size

To assess a sample of the range of petal shape and size in *Arabidopsis thaliana*, we examined 12 different natural isolates representing a broad range of genetic diversity [50](Figure S1 & S2). To quantify petal specific variation, measurements of mature (stage 13) petals from each natural isolate were normalized to those of sepals from the same isolate. Petal length was defined as the normalized length from the abscission zone to the tip of the organ; similarly, petal width was defined as the normalized value across the widest part of blade. Petal area was also normalized to corresponding sepal measurements. Petal shape, the ratio of petal length divided by width, was calculated in the same way as the leaf index, a measurement that can often be highly characteristic of a given species despite considerable environmentally-dependent variation in leaf size [51].

We observed striking and significant petal shape and size diversity. For example, the petal area in a Landsberg (La-0) ecotype flower is significantly larger than that of Columbia (Col-0); Bozen-1 petal length is significantly longer than that of Aposto-1; Rovero-1 petal shape ratio is significantly higher than that of Castelfed-4; and the Mitterberg-1 petal width significantly exceeds that of the Vancouver (Van-0) petal (Figure S2). These observations indicate that there is significant and reproducible natural variation in these petal traits, and that length, width, shape, and area can vary independently among ecotypes.

### QTL Analyses Reveal Multiple Loci Responsible for Different Aspects of Petal Form

In order to determine the genetic basis for differences in *Arabidopsis thaliana* petal forms, we measured various petal parameters in stage 13 flowers from two different RIL populations. We utilized the Columbia (Col-4)×Landsberg *erecta* (L*er*-0) RILs [37] and the Col-0×Estland-1 (Est-1) advanced intercross RILs [38]. The genomes of all of these parental lines have been fully sequenced [50]. The L*er*-0×Col-4 population has a high density marker map for the RILs [43], and the Col-0×Est-1 RILs have increased levels of recombination [38], two advantages for narrowing QTL intervals.

Approximately 20 petals and 20 corresponding sepals from 100 lines of each population and from the parental lines were photographed at floral stage 13 when petals have developed to their final shape and size [13]. Petal length, width and area were normalized to comparable data from sepals of the same line, to specifically identify loci with an effect on petal form rather than general cell-cycle control or other growth regulators. Petal shape was calculated as the ratio of petal length to petal width within the same line. Each of these petal traits displayed considerable phenotypic variation (Table S1). To determine the extent to which phenotypic variance was due to genetic variation, we calculated the broad sense heritability for each trait in each of the populations. Each of the petal traits was highly heritable (H^2^>0.89) indicating that these phenotypes are predominantly due to genetic, and not environmental, variation (Table S2).

**Figure 1:**
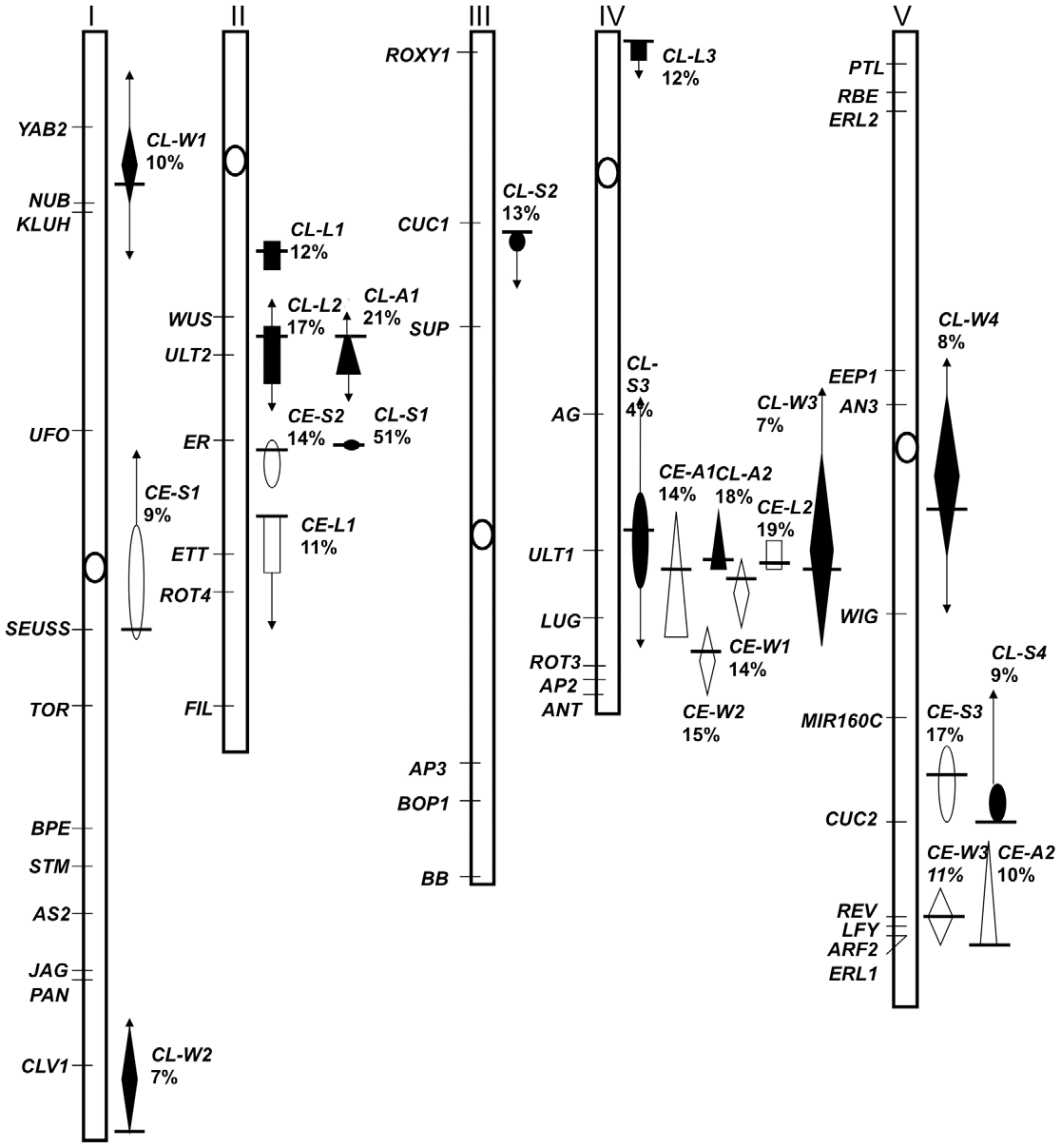
Chromosomal Locations of Quantitative Trait Loci (QTL) for Natural Variation in Petal Form in *Arabidopsis thaliana*. The chromosomal location of identified QTLs is shown on the five *Arabidopsis* chromosomes. The location of several known floral or growth regulatory genes are indicated and the centromeres marked as open circles. QTLs identified using Columbia (Col-4)×Landsberg *erecta* (L*er*) recombinant inbred lines are shown in black and named *CL*, and those identified from Columbia (Col-0)×Estland (Est) recombinant inbred lines are shown in white and named *CE*. Each QTL is indicated by a shape, with the extent of the shape indicating the 1-LOD support interval: triangle (area), rectangle (length), diamond (width), or oval (shape) and named as A, L, W, or S, respectively. The location of each QTL peak is marked with a black horizontal line and the 2-LOD support interval boundary is indicated by arrows. Where the 1-LOD and 2-LOD boundaries are the same for a QTL, no arrows are marked. Adjacent to each QTL the Percentage of Variance Explained (PVE) is noted.

To identify QTLs, a composite interval mapping approach was used employing QTL Cartographer to map QTLs to chromosomal intervals [44], [52]. A total of 23 QTLs were identified from the normalized data, 13 in the L*er*-0×Col-4 population and 10 in the Col-0×Est-1 population (Figure 1, Table 1, Figure S3). Each QTL was named in the format AB-XY, in which the letters at position AB represent the first letter of each of the parental ecotypes: C for Col (Col-0 or Col-4, depending on the context), E for Estland and L for L*er*. The letter at position X represents the petal trait, A/L/S/W for area/length/shape/width respectively, and position Y denotes the number of the particular QTL for that trait. A comparison of QTL recovery in the normalized and non-normalized datasets indicates that the normalization serves to enhance the LOD-score of a number of QTL while reducing the recovery of other, presumably non-petal-specific, QTL (Figure S4). Furthermore, the normalization can define distinct QTL for each trait; for example, for the Col-0×Est-1 data, a chromosome IV QTL for width is recovered in the normalized dataset, while a width QTL on chromosome V is recovered in the non-normalized dataset.

**Table 1:** Quantitative Trait Loci for Natural Variation in Petal Shape and Size.

QTL Name	Petal Trait	Chr	LOD Score	PVE	Effect of the Col Allele on the Trait	Map Marker Closest to the QTL peak	Map Marker at Start of the 2-LOD 95% CI	Map Marker at End of the 2-LOD 95% CI	Number of Genes in the 2-LOD Interval	Candidate Floral or Growth Regulator Genes in the 2-LOD Interval
*CL-W1*	Width	1	3.13	10	Decrease	AT1G12240	AT1G04550	AT1G18260	2094	*YAB2, NUB, KLUH*
*CE-S1*	Shape	1	3.43	9	Decrease	AT1G43560	AT1G31730	AT1G43780	1147	*SEUSS*
*CL-W2*	Width	1	2.73	7	Decrease	AT1G80580	AT1G72140	AT1G81020	1269	*PIN1, CLV1*
*CL-L1*	Length	2	5.4	12	Increase	AT2G13990	AT2G13590	AT2G14870	185	
*CL-L2*	Length	2	5.65	17	Increase	AT2G18940	AT2G17090	AT2G23910	1008	*ULT2*
*CL-A1*	Area	2	6.1	21	Increase	AT2G20020	AT2G18580	AT2G23910	795	*ULT2*
*CL-S1*	Shape	2	25.4	51	Increase	AT2G26290	AT2G25910	AT2G26820	132	*ER*
*CE-S2*	Shape	2	6.7	14	Increase	AT2G26850	AT2G26290	AT2G28940	394	*ER*
*CE-L1*	Length	2	3.1	11	Increase	AT2G31080	AT2G31080	AT2G39010	1219	*ETT, ROT4*
*CL-S2*	Shape	3	9.55	13	Decrease	AT3G17410	AT3G15510	AT3G20430	729	*CUC1*
*CL-L3*	Length	4	4.08	12	Increase	AT4G00480	AT4G00010	AT4G02940	475	
*CL-S3*	Shape	4	3.73	4	Increase	AT4G27220	AT4G17870	AT4G35080	2640	*ULT1, LEUNIG*
*CL-A2*	Area	4	6.6	18	Decrease	AT4G28950	AT4G25400	AT4G31650	936	*ULT1*
*CL-W3*	Width	4	2.39	7	Decrease	AT4G29490	AT4G17870	AT4G35080	2640	*AG, ULT1, LEUNIG*
*CE-L2*	Length	4	6.39	19	Decrease	AT4G29658	AT4G28485	AT4G30590	354	*ULT1*
*CE-A1*	Area	4	4.6	14	Decrease	AT4G29658	AT4G25640	AT4G34390	1342	*ULT1, LEUNIG*
*CE-W1*	Width	4	4.55	14	Decrease	AT4G30590	AT4G29658	AT4G34390	737	*LEUNIG*
*CE-W2*	Width	4	4.5	15	Decrease	AT4G36580	AT4G34390	AT4G38780	629	*ROT3*
*CL-W4*	Width	5	2.58	8	Decrease	AT5G35070	AT5G25900	AT5G40450	1863	*EEP1, AN3*
*CE-S3*	Shape	5	6.86	17	Increase	AT5G49570	AT5G47590	AT5G52500	727	*CUC2*
*CL-S4*	Shape	5	7.41	9	Increase	AT5G53740	AT5G44750	AT5G54230	1396	*MIR160C, CUC2*
*CE-W3*	Width	5	3.18	11	Decrease	AT5G58940	AT5G55220	AT5G63650	1224	*REV, LFY, ARF2, ERL1*
*CE-A2*	Area	5	3.79	10	Decrease	AT5G63650	AT5G55220	AT5G63650	1224	*REV, LFY, ARF2, ERL1*

The first two letters of the QTL name designate the parental lines from which the recombinant inbred lines were derived, C for Columbia, E for Estland-1, L for Landsberg *erecta*. Chr, the chromosome to which the QTl was mapped. LOD, likelihood of difference score. PVE, percentage of the variance explained (R2). CI, Confidence Interval. All petal trait measurements are normalized to comparable sepal data apart from petal shape, which is calculated as the ratio of petal length/petal width.

Many of the identified QTL intervals did not overlap with known floral or growth regulator candidate genes, therefore these QTLs reveal natural variation at genes not previously implicated in the control of petal growth or form (Figure 1). For example, none of the QTLs we identified mapped to known genes such as *RBE*, *JAG*, or *BB* that, when mutated, alter some aspect of petal form [15], [20], [53].

For the L*er*-0×Col-4 population, two QTLs were detected for petal area (totaling 39% of the phenotypic variance), three QTLs for petal length (totaling 41% of the phenotypic variance), four QTLs for petal shape (totaling 77% of the phenotypic variance) and four QTLs for petal width (totaling 43% of the phenotypic variance) (Table 1). The percentage of phenotypic variance explained by each individual L*er*-0×Col-4 QTL ranged from 4 to 51% (Table 1). For the Col-0×Est-1 populations, two QTLs were detected for petal area (totaling 24% of the phenotypic variance), two QTLs were detected for petal length (totaling 30% of the phenotypic variance), two QTLs for petal shape (totaling 40% of the phenotypic variance), and three QTLs for petal width (totaling 40% of the phenotypic variance) (Table 1). The percentage of phenotypic variance explained by each individual Col-0×Est-1 QTL ranged from 9 to 17% (Table 1). Together, these observations indicate that we have recovered a number of QTL for each trait, although it is likely that undetected QTL of smaller effect also play a role in modulating petal form in these populations.

Surprisingly, almost all the QTLs for a particular petal trait were unique to one of the two RIL populations, with the exception of a few QTLs that mapped to overlapping intervals: one interval for shape on chromosome II: *CL-S1* and *CE-S2*; one interval for area on chromosome IV: *CL-A2* and *CE-A1*; and one interval for shape on chromosome V: *CL-S4* and *CE-S3* (Figure 1, Table 1). This indicates that most of the allelic differences that affect petal shape and size between the populations we have utilized occur at different loci.

We found that growth along different directional axes can be regulated independently, as can be seen for the loci controlling petal length and width. For the three length and four width QTLs for L*er*-0×Col-4, none of these different petal trait QTL intervals overlap, and for the two length and three width QTLs for Col-0×Est-1, only one set of these QTL intervals overlap, *CE-W1* and *CE-L2* on chromosome IV (Figure 1). In fact, this region on chromosome IV contained the greatest concentration of overlapping QTLs for multiple petal traits for both sets of RILs (Figure 1). As this QTL interval overlapped with the *ULTRAPETALA1 (ULT1)* locus, a known floral meristem regulator with a role in petal development [54], we tested whether *ULT1* might be responsible for this QTL. Among the ecotypes used in this study, none showed any polymorphic sites within the *ULT1* coding region or in the 2 kilobases upstream of the start codon (http://signal.salk.edu/atg1001/3.0/gebrowser.php). Furthermore, the *ult1-3* T-DNA insertional mutation in the first exon of *ULT1* [40] showed no significant effect on petal form (Figure S5). Together, these data suggest that allelic variation at *ULT1* is not responsible for this QTL.

### Allelic Variation at the *ERECTA* Locus Regulates Petal Shape

Overlapping QTLs recovered in both populations for the same trait represented potential loci with considerable allelic diversity contributing to the quantitative petal trait. We concentrated our initial mapping efforts on the *CL-S1* QTL on chromosome II, a QTL of large effect which alone explained 51% of the petal shape variation in the L*er*-0×Col-4 RIL population (Table 1) and an interval that also overlaps with *CE-S2* (Figure 1).

**Figure 2:**
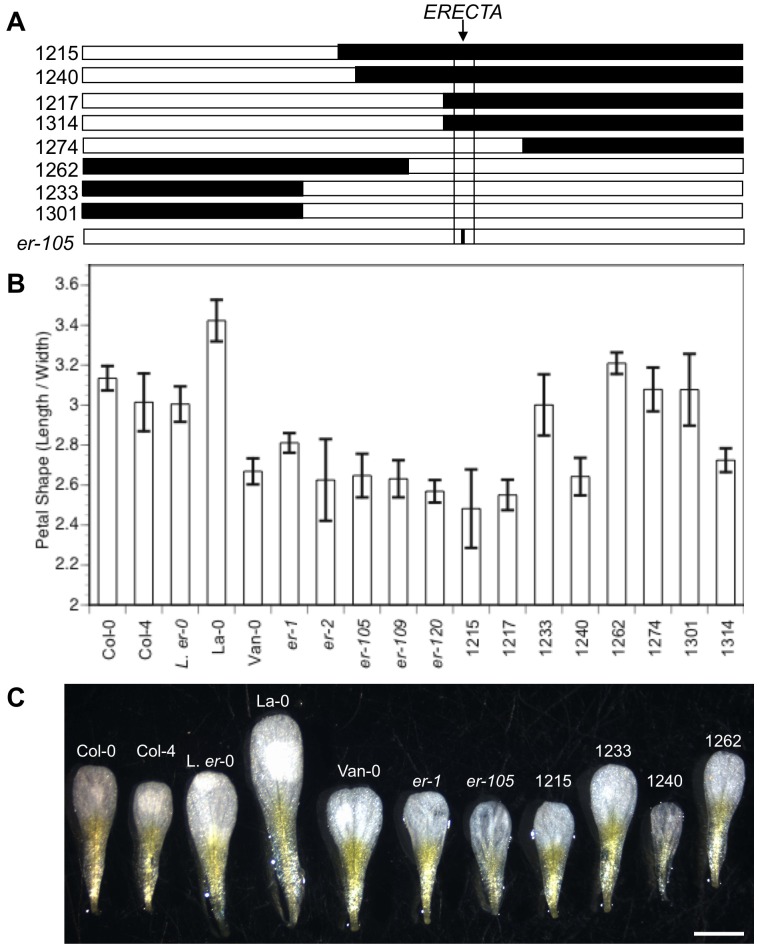
*ERECTA* Corresponds to the Petal Shape Quantitative Trait Locus (QTL) *CL-S1* on Chromosome II. (**A**) Chromosome II diagram showing introgression lines and mutants used for QTL fine mapping. The Stepped Aligned Inbred Recombinant Strain (STAIRS) introgression lines are numbered and their L*er* -0 introgressions are drawn to scale in black and the otherwise entirely Columbia (Col-4) background is shown in white. The location of the *CL-S1* 2-LOD interval is drawn to scale with the boundaries marked as black horizontal lines, and the *er* mutants used, such as *er-105*, have an *er* mutation in an otherwise entirely Columbia (Col-0) background (apart from L*er*-0 which is an *er* mutant in a Landsberg background). (**B**) Histogram showing shape measurements of stage 13 petals using STAIRS, natural isolates, and *er* mutants. Error bars indicate 95% confidence intervals. For each line a minimum of 20 petals were scored. (**C**) Stage 13 representative petal images. Scale bar = 1 mm.

To confirm and fine-map *CL-S1*, we used a set of mapping tools known as Stepped Aligned Inbred Recombinant Strains (STAIRS) [48]. The STAIRS contain L*er* genome introgressions in an otherwise entirely Col-0 background, such that individual lines contain a single chromosome with a nested L*er* introgression. We tested a set of STAIRS with different L*er* introgression boundaries on chromosome II (Figure 2). In agreement with the QTL predictions that a Col allele at the *CL-S1* locus would increase the shape ratio value (Table 1), all of the lines that contained a L*er* introgression encompassing the QTL interval *CL-S1* (lines 1215, 1217, 1240, and 1314) showed a significant reduction in the petal shape ratio value compared with Col, while those that had L*er* introgressions elsewhere on chromosome II (lines 1233, 1262, 1274, and 1301) did not show a significant petal shape ratio change compared with the Col parental line (Figure 2).

To identify the gene responsible for *CL-S1*, we focused on the 132 genes that mapped within the 2 likelihood of odds (LOD) QTL interval (Table 1); these included the candidate gene *ERECTA* (*ER*) that has previously been identified as a central growth regulator in a variety of plant developmental contexts [55]. *ER* encodes a leucine-rich repeat receptor-like serine/threonine kinase that has been implicated in the regulation of cell proliferation and been suggested to act as a plant growth factor receptor [39], [56], [57].

To determine if *ER* might be the gene responsible for *CL-S1*, we examined five independent strong *er* mutations in an otherwise Col-0 background, including several nulls. Each of these *er* mutations alone was sufficient to markedly affect petal shape as compared with the Col-0 background (Figure 2). The natural isolate Vancouver (Van-0) displays the compact morphology characteristic of an *er* mutant phenotype and contains an *er* null allele due to a substitution at the start codon [58]. Van-0 petals had a significantly smaller petal length∶width ratio as compared with Col-0 (Figure 2). In addition, we examined the original Landsberg strain (La-0) from which L*er* was subsequently isolated by X-irradiation of a population of Landsberg seed [59]. L*er* has a missense mutation within the kinase domain of the ER protein [39]. La-0 petals displayed a large petal shape index (larger than Col-0), however, the L*er*-0 mutation in that Landsberg background was sufficient to significantly decrease the petal shape index (Figure 2). All these results are consistent with the *CL-S1* QTL regulating petal shape corresponding to *ER*. Interestingly, a direct comparison of the L*er*-0 and the Col-4 parental lines does not show a significant petal shape difference (Figure 2) presumably because of other counterbalancing allelic differences outside the *CL-S1* region.

We also tested the ability of an L*er* line containing a Col-0-derived ER transgene, *ER*-L*er* [58], to confer a petal shape phenotype more similar to that of the Col-0 ecotype. The introduced transgene partially complemented the *er* petal shape phenotype, such that the *ER*-L*er* petal shape was more similar to that of the Col-0 ecotype (Figure S6). In keeping with our identification of at least six other loci controlling petal shape differences, the lack of complete rescue by the transgene supports the idea that allelic differences between Col-0 and L*er* at other genes, whose products may interact with ER, can also contribute to petal shape differences.

A QTL for shape, *CE-S2*, was also found in an overlapping interval to *CL-S1* in our other mapping population. However, *CE-S2* does not appear to be caused by a polymorphism in the Estland *ER* coding sequence (Table S3) or in the three kilobases upstream of the start codon (http://signal.salk.edu/atg1001/3.0/gebrowser.php). Furthermore, Estland plants do not show the highly compact morphology characteristic of an *er* mutation [39], suggesting that Estland plants are not compromised for *ER* function.

### Differences in Petal Shape Caused by Different *ER* Alleles Are Due to Alterations in Cell Proliferation

**Figure 3:**
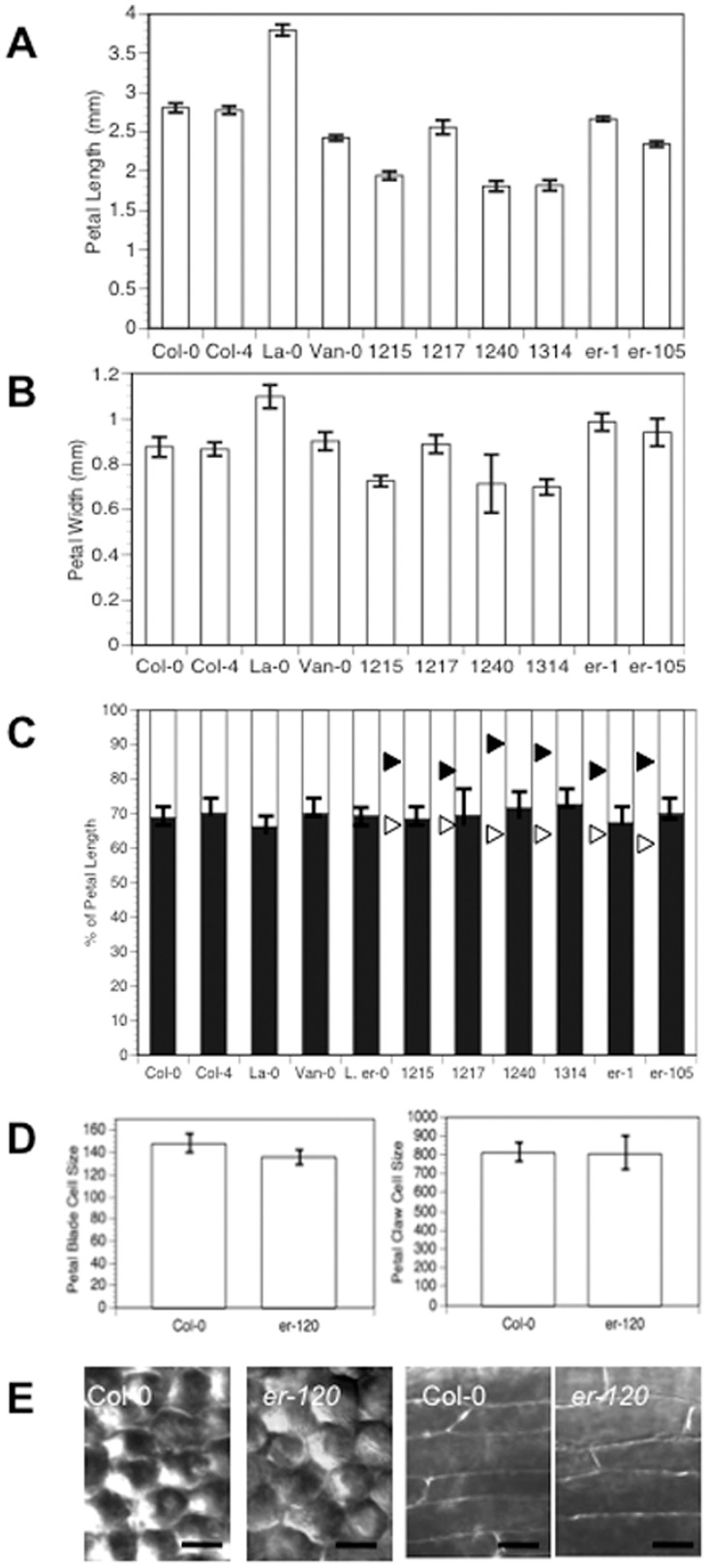
Petal Shape Differences Associated with Different *ER* Alleles Are Caused by Differences in Cell Proliferation. (A) and (B) Histograms showing stage 13 petal lengths and widths. The error bars for all graphs represent the 95% confidence intervals. (C) Stacked bar chart showing the mean % of petal length for blade (white) and claw (black) for the lines indicated. The black arrowhead indicates where the blade/claw boundary would be located if the length change for that line was just due to changes in the blade, and the white arrowhead indicates where the blade/claw boundary would be located if the length change for that strain was just due to changes in the claw. (D) Average cell size in microns^2^ was calculated for the blade and the claw. A minimum of three regions on three petals were sampled for each measurement. (E) Sample petal stage 13 blade (left) and claw (right) cells for the genotypes indicated. Scale bars = 20 µm.

To further define the processes that were contributing to the differences in petal shape, measurements were made of petal length and width of STAIRS lines and *er* mutants that have a petal shape change relative to Col-0. Petal shape differences appear to be predominantly driven by changes in organ length (Figure 3). The mean width of the petals only varies by about 0.1 mm but petal length variations can be as much as approximately 1 mm (Figure 3).

To determine if the length was being preferentially affected in the blade (the distal area of the petal with white epidermal cells) or the claw (the green basal area of the petal) or across both regions, measurements were made of the blade and claw lengths in STAIRS lines, *er* mutants, and several natural isolates. Despite a wide range of petal lengths, the blade to claw length ratio remained equivalent in all lines tested (Figure 3). These observations indicate that petal length in these lines is coordinately controlled along the entire organ axis.

To determine if the differences in petal shape were due to differences in cell number or cell size, petal cell size was assessed in the blade and claw in Col-0 and *er-120*, a strong EMS-induced *er* allele in the Col-0 background (Figure 3). Average cell size was calculated by assessing the number of cells in a given unit area of the blade or claw (see Methods). No significant difference in average cell size was apparent when comparing these two genotypes (Figure 3). Therefore the differences in petal shape must be due to differences in cell proliferation that result in a final difference in cell numbers. Furthermore, these alterations in cell proliferation appear to be occurring in a coordinated fashion along the entire length of the petal.

## Discussion

We have analyzed the genetic basis for some of the natural variation regulating petal form in *Arabidopsis thaliana* and identified 23 loci for quantitative traits regulating aspects of petal shape and size. Through these analyses, we demonstrated that the genetic control of different parameters, such as petal length and width, can be uncoupled. We also showed that allelic variation at the *ER* locus can result in quantitative differences in petal shape, due to differences in the regulation of cell proliferation across the entire length of the petal.

### Genetic Control of Petal Shape and Size

Our analyses of natural variation for petal form, and the identification of QTLs underlying different aspects of petal morphology, provide a framework for better understanding the genetic basis of these traits. By normalizing the petal parameters measured to those of sepals, we were able to identify QTL affecting the morphology of an individual floral organ type. Normalizing these data has allowed us to identify a number of new loci that do not correspond to previously identified regulators of petal growth or form (Figure 1). However, such normalization may introduce other biases in recovery of relevant QTL since variation in sepal shape and size is also segregating in the RIL populations. However, the use of composite interval mapping which employs a regression analysis presumably minimized the effects of segregating sepal variation on the identification of a petal QTL. The number of loci we recovered as affecting petal form is likely to be an underestimate; almost all of the QTLs identified for a particular trait were only recovered in a single RIL population, suggesting that there is still substantial variation to be uncovered.

Despite the lack of recovery of coincident QTL among the two RIL populations assayed, the underlying genetic architecture for the different petal traits that we scored were quite similar. For example, for width, we recovered four moderate effect L*er*-0×Col-4 QTL (each explaining 7–10% of the variance) and three moderate effect Col-0×Est QTL (each explaining 11–15% of the variance). We did not detect any obvious clustering of QTL for any trait, although about one third of the QTL we recovered mapped to the bottom arm of chromosome IV suggesting there may be some correlation across traits. Furthermore, it is clear that although many growth regulators have been identified that coordinately control aspects of both leaf and floral organ size [5], there are a considerable number of loci that act in an organ specific manner and are presumably regulated by organ identity genes. We identified QTL that independently affected length, width, shape and area, indicating that these different aspects of petal morphology can be controlled separately and supporting the idea of developmental modularity in the regulation of plant growth. This is also consistent with previous studies that have identified distinct QTL affecting leaf versus floral size [35], [60], [61].

The results of our study can be compared to that of Juenger et al. [35], who carried out a similar analysis to identify QTL that affected petal length and width in *Arabidopsis thaliana*. Their analysis differed from ours in that they utilized a L*er* X Cvi recombinant inbred population, and did not normalize their petal measurements. They identified six petal length QTL and two petal width QTL in their mapping population. With the exception of a petal length QTL mapping in the vicinity of the *ER* locus, none of the QTLs recovered in that study overlapped with length or width QTL identified in either of our mapping populations. Furthermore, the QTL mapping to the *ER* locus identified in the Juenger et al (2005) study only explained 17.7% of the variance as compared to the 51% of the variance explained by the *ER* locus in our mapping study. This suggests at least two possibilities. It is possible that allelic differences among the different accessions used in these analyses vary to such a great extent that each analysis is only capturing a small proportion of the actual allelic diversity present for loci that affect these traits. Such allelic diversity could also explain the differences in the degree to which each identified locus explains the variance. Alternatively, it may be that the lack of normalization in the Juenger et al. (2005) study resulted in identification of loci that have more general roles in regulating organ form.

We also identified distinct QTL affecting petal shape, which was measured as a ratio of length to width. Surprisingly, the shape QTL were not generally coincident with either length or width QTL recovered in our analyses, despite being calculated on the basis of these parameters. This suggests that some QTL affect length and width oppositely, such that variation in either length or width may not be statistically significant, but in combination can result in significant variation in shape. These observations suggest that some loci act to regulate both length and width, while others affect each parameter independently. Furthermore, subtle differences in the expression levels of component genes involved in a regulatory network can culminate in morphological differences among closely related taxa (eg. [62]). As such, it is likely that the genetic context of each RIL has influenced recovery of the QTL we have identified.

### *ERECTA* is a Key Petal Shape Regulator

*ER* has been identified in *Arabidopsis* genome-wide studies as one of six major hotspots for phenotypic variation [63]–[65]. We have shown that natural variation at the *ER* locus is one factor responsible for differences in petal shape, due to effects on petal cell proliferation along the entire extent of the petal.

*ER* has previously been identified as having a pleiotropic effect on many *Arabidopsis* growth phenotypes, including stomata development, leaf size, hypocotyl elongation, pedicel development, and ovule differentiation [55]. However, the role of *ER* in modulating growth appears to be distinct in different tissues, and may reflect differential interactions of the ER LRR receptor-like kinase with extracellular signals. For instance, *ER* mediates hypocotyl growth in response to light cues [66] and hyponastic growth of petioles in response to ethylene [58], [67]. In leaves, *ER* has been shown to control both epidermal cell number as well as cell size [68]. This is in contrast to our observation that, in petals, variation at the *ER* locus has little effect on cell size and appears to act predominantly through modulating cell proliferation. The distinction between the effects of allelic variation at *ER* on leaf versus petal cell dynamics could reflect the robustness of petal cell size towards variation at *ER*. As petal epidermal cells have a distinctive cellular morphology that is of ecological significance in some species [69], it may be that epidermal cell size is more highly constrained than cell number in this organ.

Petals arise through an initial phase of cell division, followed by a basipetal progression of cell-cycle arrest and subsequent cell expansion and differentiation [15], [33], [70]. The transition zone from cell division to cell expansion is known as the arrest front. One possibility is that *ER* may be acting to regulate the timing of the initiation of the arrest front. It is unlikely that *ER* acts through modulating the speed of the arrest front progression, as we observe corresponding shifts in cell number in both petal blade and petal claw for different *er* alleles. As ER likely acts as a receptor for extracellular signals [39], it is possible that ER acts as a receptor for a mobile signal that regulates arrest front progression at the organ level.

*ERECTA-LIKE 1 (ERL1)* and *ERL2* encode receptor-like kinases that act redundantly with *ER* in some developmental processes [57]. *ERL1* and *ERL2*, like *ER*, are expressed in developing floral primordia including petals [57]. Some of the QTL we identified may correspond to these loci or other components of the ER signaling pathway. The *ERL1* locus overlaps with the *CE-A2* and *CE-W3* intervals, although we did not identify any shape QTL within this region.

### Other loci regulating petal shape and size

It is surprising that so few of the QTL recovered in this study map to regions delimiting previously defined petal regulatory genes. This contrasts with the recovery of QTL underlying vernalization and flowering time variation in *Arabidopsis thaliana*, in which much of the variation across several accessions appears to be due to allelic differences at two previously well-characterized loci, *FLC* and *FRI* [71].

During leaf growth, there is stage specific regulation of competence to respond to specific developmental cues; for example, expression of expansin is most effective during the phase of maximum leaf growth, while expression at other times has relatively little effect on cell expansion [72]. These studies and others indicate that the coordination of growth and gene expression must rely on regulatory factors that act in a context-dependent manner. QTL studies such as the one reported here may be one way to identify new genetic regulators that modulate spatial and temporal aspects of petal growth.

## Supporting Information

Figure S1 **Petal Variation in** ***Arabidopsis thaliana*** **Natural Isolates and Recombinant Inbred Lines.** (A) Sample petal (top) and sepal (bottom) from the same stage 13 bud from parental and offspring RILs for Columbia (Col-4)×Landsberg *erecta* (L*er*). Scale bar = 1 mm. (B) Sample petal and sepal from the same stage 13 bud from each of the natural isolates indicated. Scale bar = 1 mm. (C) and (D) Whole stage 13 floral buds. Scale bar = 3 mm.
(TIFF)

Figure S2 **Petal Morphological Variation in Some** ***Arabidopsis thaliana*** **Natural Isolates.** (A) Petal area normalized to sepals. (B) Petal length normalized to sepals. (C) Petal shape (length/width). (D) Petal width normalized to sepals. For each graph the error bars represent 95% confidence intervals and a minimum of 12 petals and 12 sepals were scored for each line.
(TIF)

Figure S3 **Quantitative Trait Loci Analysis of Natural Variation in** ***Arabidopsis thaliana*** **Petal Morphology.** Panels show the logarithm of differences (LOD) profile for the QTL results for each trait per chromosome with the additive effect trace below. Chr = chromosome. The top graph in each panel is for the Col-4×L*er* recombinant inbred lines (RILs) and the bottom graph is for the Col-0×Est RILs. (**A**) Petal length normalized to sepals. (**B**) Petal width normalized to sepals. (**C**) Petal area normalized to sepals. (**D**) Petal shape (petal length/width).
(TIF)

Figure S4 **Quantitative Trait Loci Analysis of Natural Variation in** ***Arabidopsis thaliana*** **Petal Morphology.** Panels show the logarithm of differences (LOD) profile for the QTL results for each trait per chromosome with the additive effect trace below. Chr = chromosome. The top graph in each panel is for the Col-4×L*er* recombinant inbred lines (RILs) for petal data and the bottom graph is for the Col-0×Est RILs. The black trace is for petal data normalized to sepals and the red trace is for petal data not normalized to sepals. (**A**) Petal length. (**B**) Petal width. (**C**) Petal area.
(TIF)

Figure S5 ***ULTRAPETALA1*** **Does Not Alter Petal Area or Length.** (**A**) and (**B**) Histograms of measurements of stage 13 petals normalized to sepals for Col-0 (n = 36 petals), and *ult1-3* (confirmed Salk line SALK_074642C with a T-DNA insertion in a Col-0 background that displays *the ult1* mutant phenotype) (n = 40 petals). Error bars represent 95% confidence intervals.
(TIFF)

Figure S6 **The ER allele partially restores the petal shape phenotype in the L** ***er*** **ecotype.** Histogram showing petal shape measurements for Col-0, L*er* and *Ler-ER* lines. Error bars represent 95% confidence intervals, n = 23 to 39 petals per line tested.
(TIF)

Table S1 **Phenotypic Range in the QTL Analysis Study.**
(DOCX)

Table S2 **Broad Sense Heritability (H^2^).**
(DOCX)

Table S3 **Natural Variation in the ERECTA Gene and Promoter.**
(DOCX)
