# Supplementary figures and images for: Natural Variation Identifies Multiple Loci Controlling Petal Shape and Size in Arabidopsis thaliana
Source: PLoS One. 2013 Feb 13;8(2):e56743. doi: 10.1371/journal.pone.0056743 (PMC3572026; doi:10.1371/journal.pone.0056743)

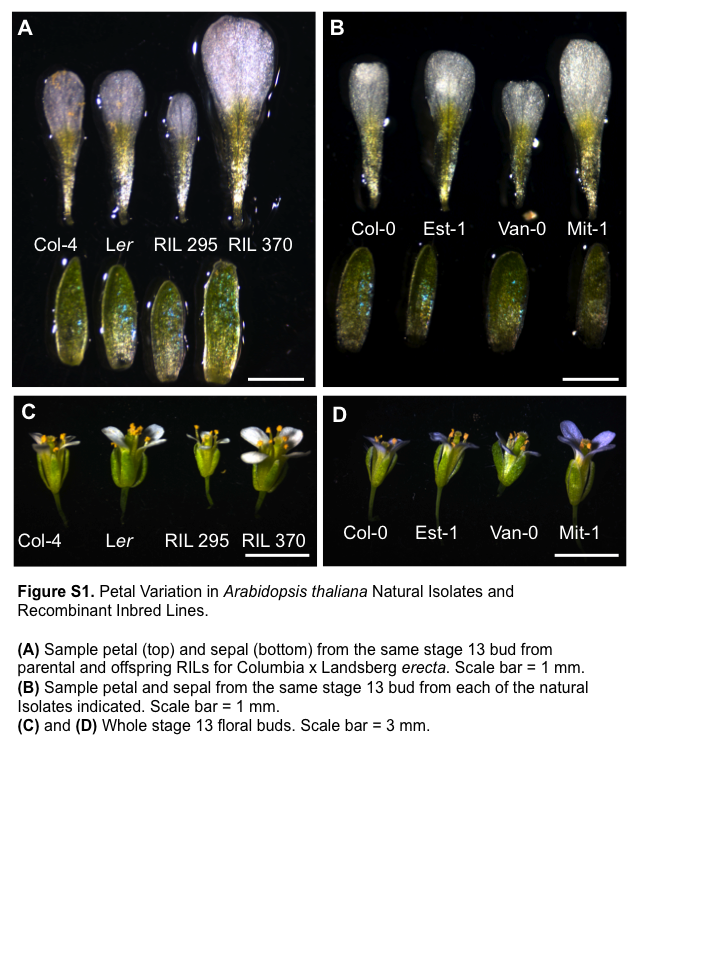

Supplement: Figure S1 — Petal Variation in Arabidopsis thaliana Natural Isolates and Recombinant Inbred Lines. (A) Sample petal (top) and sepal (bottom) from the same stage 13 bud from parental and offspring RILs for Columbia (Col-4)×Landsberg erecta (Ler). Scale bar = 1 mm. (B) Sample petal and sepal from the same stage 13 bud from each of the natural isolates indicated. Scale bar = 1 mm. (C) and (D) Whole stage 13 floral buds. Scale bar = 3 mm. (TIFF) [file pone.0056743.s001.tiff]

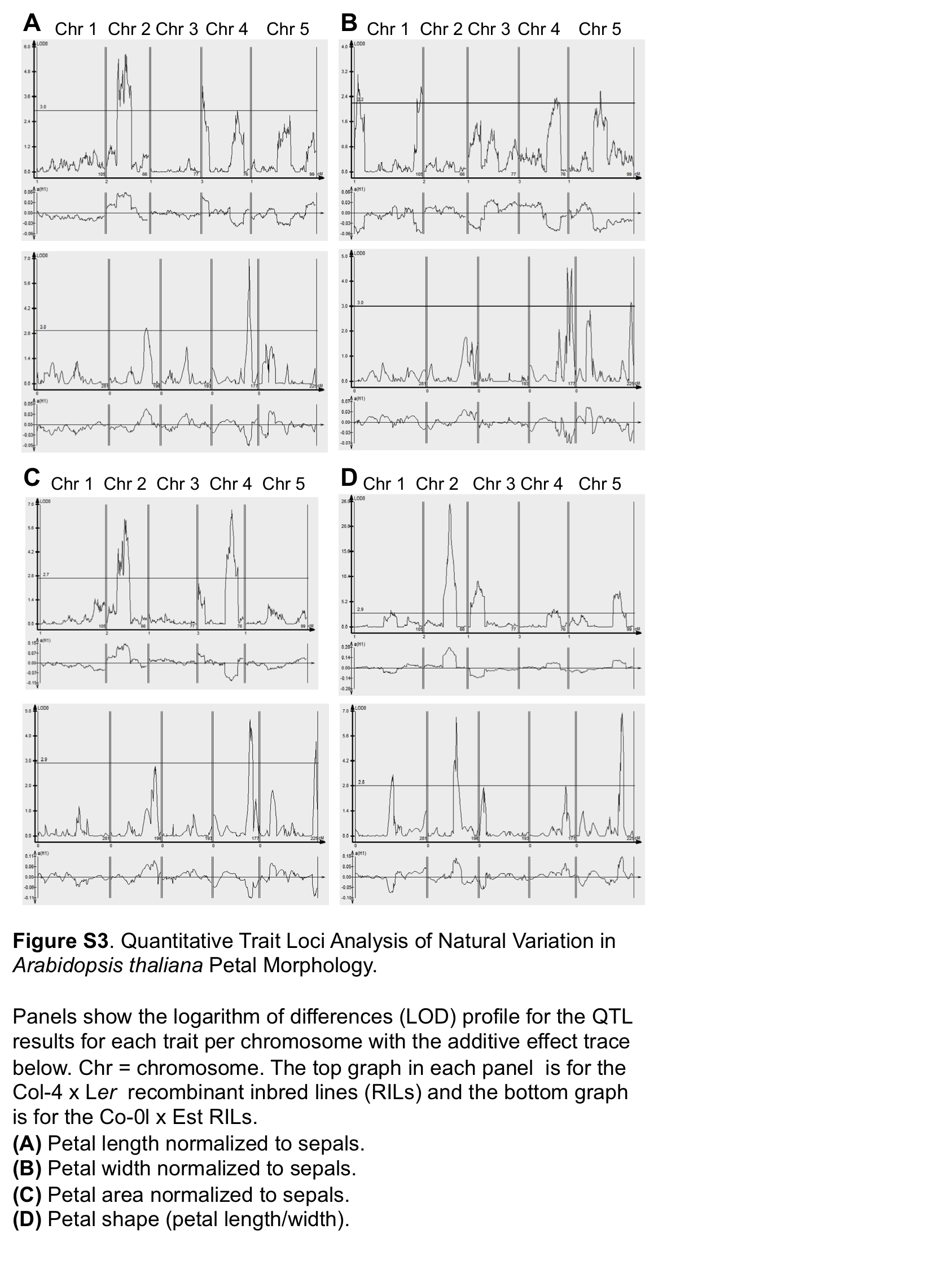

Supplement: Figure S3 — Quantitative Trait Loci Analysis of Natural Variation in Arabidopsis thaliana Petal Morphology. Panels show the logarithm of differences (LOD) profile for the QTL results for each trait per chromosome with the additive effect trace below. Chr = chromosome. The top graph in each panel is for the Col-4×Ler recombinant inbred lines (RILs) and the bottom graph is for the Col-0×Est RILs. (A) Petal length normalized to sepals. (B) Petal width normalized to sepals. (C) Petal area normalized to sepals. (D) Petal shape (petal length/width). (TIF) [file pone.0056743.s003.tif]

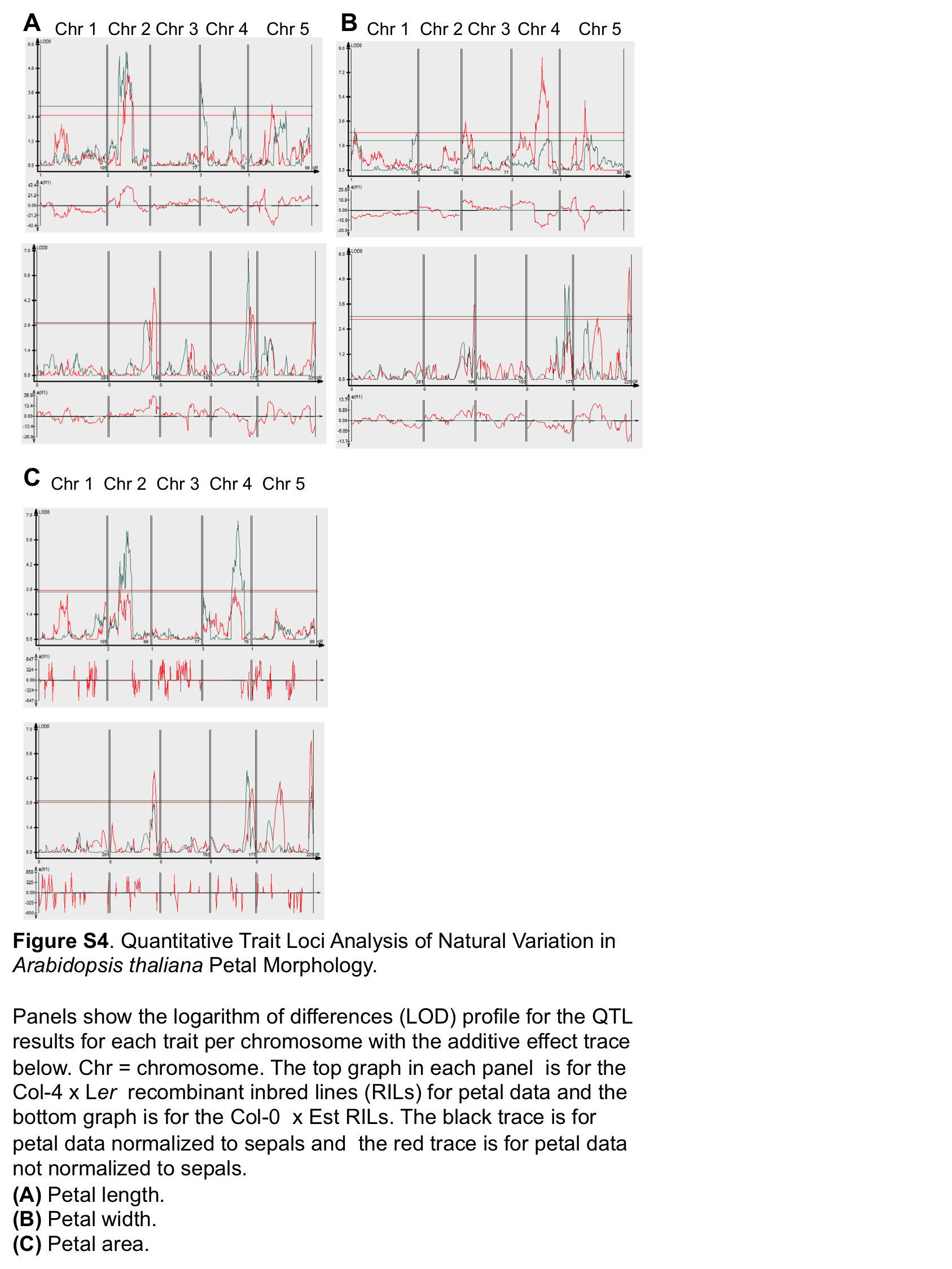

Supplement: Figure S4 — Quantitative Trait Loci Analysis of Natural Variation in Arabidopsis thaliana Petal Morphology. Panels show the logarithm of differences (LOD) profile for the QTL results for each trait per chromosome with the additive effect trace below. Chr = chromosome. The top graph in each panel is for the Col-4×Ler recombinant inbred lines (RILs) for petal data and the bottom graph is for the Col-0×Est RILs. The black trace is for petal data normalized to sepals and the red trace is for petal data not normalized to sepals. (A) Petal length. (B) Petal width. (C) Petal area. (TIF) [file pone.0056743.s004.tif]

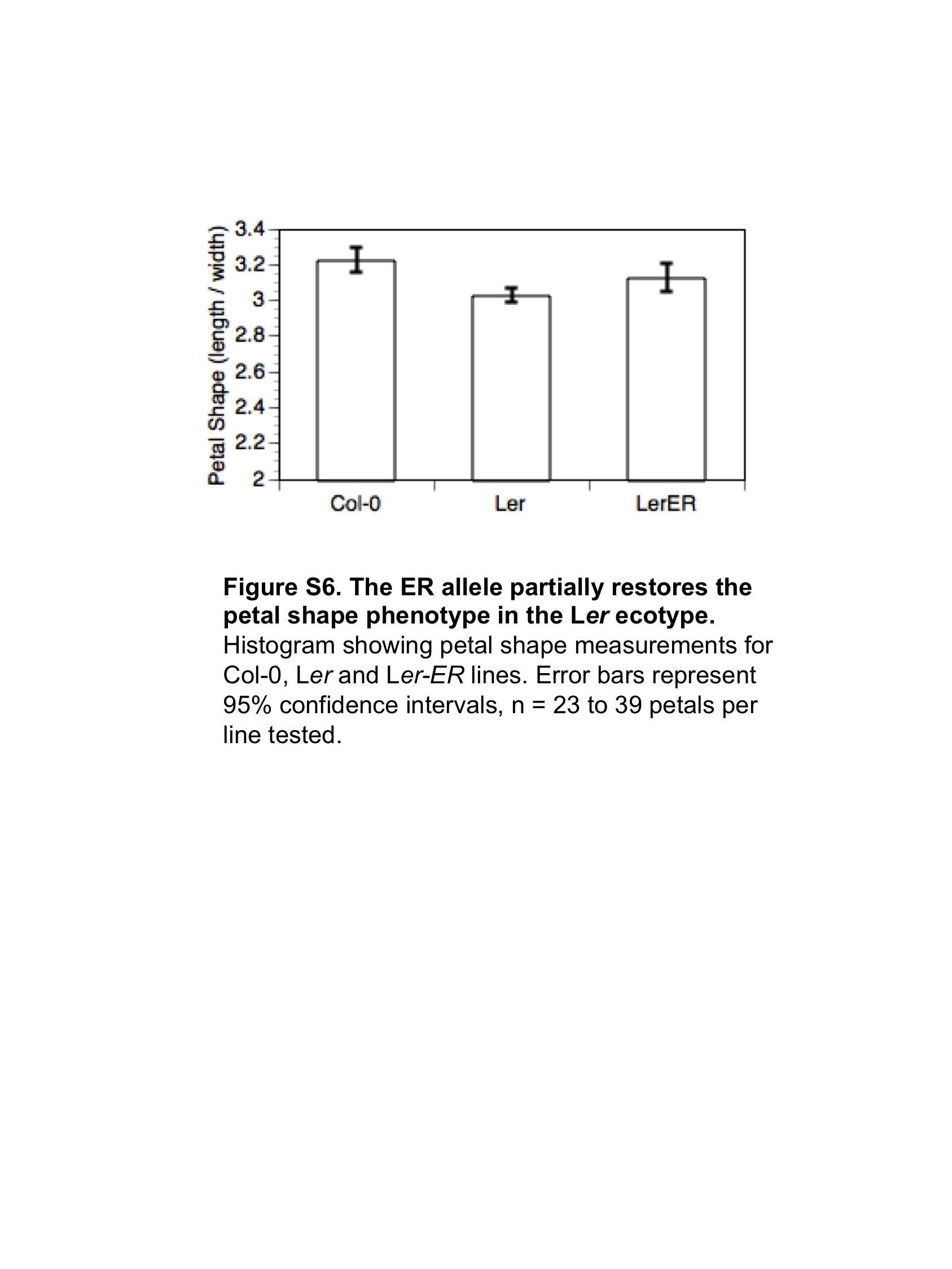

Supplement: Figure S6 — The ER allele partially restores the petal shape phenotype in the L er ecotype. Histogram showing petal shape measurements for Col-0, Ler and Ler-ER lines. Error bars represent 95% confidence intervals, n = 23 to 39 petals per line tested. (TIF) [file pone.0056743.s006.tif]
