# Supplementary material for: Natural Variation Identifies Multiple Loci Controlling Petal Shape and Size in Arabidopsis thaliana
Source: PLoS One. 2013 Feb 13;8(2):e56743. doi: 10.1371/journal.pone.0056743 (PMC3572026; doi:10.1371/journal.pone.0056743)
Supplement: Table S1 — Phenotypic Range in the QTL Analysis Study. (DOCX) [file pone.0056743.s007.docx]

**Table S1.** Phenotypic Range in the QTL Analysis Study

| Parents for the RILs | Petal Trait Analyzed | RILs Mean | RIL Minimum | RIL Maximum | Col Mean | L*er*-0 or Est-1 Mean |
| --- | --- | --- | --- | --- | --- | --- |
| Col-4 × L*er*-0 | Area | 1.62 | 1.05 | 2.65 | 1.64 | 1.46 |
| Col-4 × L*er*-0 | Length | 1.62 | 1.29 | 1.89 | 1.63 | 1.52 |
| Col-4 × L*er*-0 | Shape | 3.05 | 2.23 | 4.15 | 3.4 | 2.99 |
| Col-4 × L*er*-0 | Width | 1.36 | 1.05 | 1.95 | 1.33 | 1.33 |
| Col-0 × Est-1 | Area | 1.84 | 1.04 | 2.57 | 1.6 | 2.3 |
| Col-0 × Est-1 | Length | 1.56 | 1.31 | 1.8 | 1.5 | 1.62 |
| Col-0 × Est-1 | Shape | 2.86 | 2.28 | 3.48 | 3.18 | 2.7 |
| Col-0 × Est-1 | Width | 1.51 | 1.19 | 1.89 | 1.47 | 1.88 |

Values represent petal traits normalized to sepals. For Col-0 × L*er*-0 population, n = 490 buds; for Col-0×Est-1 population, n = 565 buds.
