# Supplementary material for: Natural Variation Identifies Multiple Loci Controlling Petal Shape and Size in Arabidopsis thaliana
Source: PLoS One. 2013 Feb 13;8(2):e56743. doi: 10.1371/journal.pone.0056743 (PMC3572026; doi:10.1371/journal.pone.0056743)
Supplement: Table S2 — Broad Sense Heritability (H2). (DOCX) [file pone.0056743.s008.docx]

**Table S2.** Broad Sense Heritability (H^2^)

| RIL Population | Petal Area | Petal Length | Petal Width | Petal Shape |
| --- | --- | --- | --- | --- |
| Col-0 × Est-1 | 0.94 | 0.94 | 0.89 | 0.90 |
| Col-0 × L*er*-0 | 0.94 | 0.89 | 0.96 | 0.96 |

H^2^, the ratio between the genetic variance and the total phenotypic variance (between and among RILs) was calculated using ANOVA (see Materials and Methods).
