# Supplementary material for: Natural Variation Identifies Multiple Loci Controlling Petal Shape and Size in Arabidopsis thaliana
Source: PLoS One. 2013 Feb 13;8(2):e56743. doi: 10.1371/journal.pone.0056743 (PMC3572026; doi:10.1371/journal.pone.0056743)
Supplement: Table S3 — Natural Variation in the ERECTA Gene and Promoter. (DOCX) [file pone.0056743.s009.docx]

**Table S3.** Natural Variation in the ERECTA Gene and Promoter

| Location of Coding Sequence Polymorphism | Ecotype | Nucleotide and Codon Change | Codon Change Consequence |
| --- | --- | --- | --- |
| Chr II: 1,217,733 (exon 19) | Est-1 | A:G (ACT to ACC) | Amino acid 464 remains Thr |
| Chr II: 1,217,733 (exon 19) | L*er*-1 | A:G (ACT to ACC) | Amino acid 464 remains Thr |
| Chr II: 1,217,172 (exon 22) | Est-1 | A:G (GGT to GCC) | Amino acid 536 remains Gly |
| Chr II: 1,217,172 (exon 22) | L*er*-1 | A:G (GGT to GCC) | Amino acid 536 remains Gly |
| Chr II: 1,216,211 (exon 26) | L*er*-1 | A:T (ATA to AAA) | Amino acid 750 Ile becomes Lys |

The location and nature of polymorphisms in *Arabidopsis thaliana* Estland and Landsberg *erecta* ecotypes compared to the Columbia reference accession.
